# Music and speech time perception of musically trained individuals: The effects of audio type, duration of musical training, and rhythm perception

**DOI:** 10.1177/17470218231205857

**Published:** 2023-10-28

**Authors:** Miria N Plastira, Michalis P Michaelides, Marios N Avraamides

**Affiliations:** 1 Department of Psychology, University of Cyprus, Nicosia, Cyprus; 2 CYENS Centre of Excellence, Nicosia, Cyprus

**Keywords:** Time perception model, Music Time Perception, Speech Time Perception, musical training

## Abstract

The perception of time is a subjective experience influenced by various factors such as individual psychology, external stimuli, and personal experiences, and it is often assessed with the use of the reproduction task that involves individuals estimating and reproducing the duration of specific time intervals. In the current study, we examined the ability of 97 musically trained participants to reproduce the durations of temporal intervals that were filled with music or speech stimuli. The results revealed a consistent pattern of durations being underestimated, and an association was observed between the duration of musical training and the level of accuracy in reproducing both music and speech tracks. In addition, speech tracks were overall reproduced more accurately, and as longer, than music tracks. Structural models suggested the presence of two, highly correlated, dimensions of time perception for speech and music stimuli that were related to the duration of musical training, but not with self-reported rhythm perception. The possible effects of arousal and pleasantness of stimuli on time perception are discussed within the framework of an internal clock model.

Although people typically use language to communicate with each other and to share their emotions, thoughts, and intentions, they can still communicate and express their selves via other means. Music is one such means (Glennie & MacDonald, 2005).

Although music and speech are systems with discrete representations, social functions, and structure Asaridou & McQueen, 2013(;esson et al., 2011), they both rely on auditory signals with common parameters, e.g., frequency, duration, intensity, and timbre (Besson et al., 2011). Yet, it is not clear whether we process speech and music stimuli using the same cognitive and neural mechanisms. On one hand, neuroimaging studies show that common brain regions, such as the Brodmann Area 47 and the motor cortex, are activated by both speech and music stimuli (Brown et al., 2006; Levitin & Menon, 2003; Rimmele et al., 2021). On the other hand, other studies report brain asymmetry, with the left and right auditory regions processing speech and melodic content, respectively (Albouy et al., 2020; Sidtis & Volpe, 1988).

If music and speech processing share common mechanisms, one would expect that expertise in one of the two domains (e.g., being an expert musician) would influence the processing of the acoustic properties of sounds in the other domain (e.g., perceiving linguistic stimuli). Indeed, a study by Yu et al. (2017) showed that musically trained participants outperformed non-trained individuals in music tasks that assessed the abilities of melodic and rhythmic analysis and in linguistic tasks that assessed semantic processing abilities. Furthermore, the spontaneous activation of the bilateral precentral gyrus in resting fMRI data correlated with the performance on both melodic analysis tasks and semantic processing tasks, supporting the notion that music and language depend on common brain mechanisms. In line with this conclusion are results from other studies showing that, compared with non-musicians, musically trained individuals are better at processing pitch and detecting pitch changes in linguistic stimuli either in their native language (Schön et al., 2004) or in a foreign language (Marques et al., 2007).

A study by Marie et al. (2012) assessed not only the effects of musical, but also of linguistic expertise, on the detection of auditory changes. In one experiment, Finnish-speaking and French-speaking participants with no-music education, as well as French-speaking musicians, were compared in a task for which they were instructed to watch a silent movie while ignoring the harmonic sounds of different durations (27 ms or 52 ms), frequencies, and intensities that were played. During this task Electroencephalography (EEG) data were recorded. In another experiment, participants were asked to pay attention to the sounds and report frequency and duration deviations from standard stimuli (of a 75 ms duration). Overall, results from the two experiments showed that the musically trained French and the non-musically trained Finnish participants were better at detecting the duration changes, compared with the non-musically trained French (Marie et al., 2012). The authors attributed the advantage of the Finish participants to the fact that in the Finnish language duration is used phonologically and the meaning of otherwise similar words changes based on phoneme duration. That is, Finnish words include various timing patterns that French words do not, and the experience (of the Finish-speaking participants) with these patterns seems to improve timing skills that generalise to other types of stimuli. As these improved timing skills were also related to the musical expertise, because musically trained French outperformed the non-trained French the authors claimed that the comparable effects of linguistic and musical expertise, support the notion that the duration of speech and music are processed by the same mechanisms (Marie et al., 2012). Overall, past studies suggest that common cognitive and neural networks underlie the perception of speech and music stimuli, despite some evidence for the involvement of specialised areas of the brain in each type of stimulus (Albouy et al., 2020; Sidtis & Volpe, 1988). Extending past research, the goal of the present study is to explore whether time perception differs for music and speech stimuli by comparing directly the duration estimates for both types of stimuli in the same task and with a large sample of musically trained participants. In contrast to the short (in the range of milliseconds) stimuli used by Marie et al., (2012), here we use durations that are in the range of seconds, with the rationale that in everyday life people are not exposed to music that has duration in the range of milliseconds. Plus, when people communicate, they are typically exposed to speech for short, but not that brief, **periods** of time.

In the present study, we investigate whether biases in the duration estimates for these two types of audio stimuli can be accounted by a single general dimension of time perception, or instead by distinct music-specific and speech-specific time perception dimensions. In addition, we investigate whether the extend of one’s musical training is linked to time estimation performance, either for intervals containing music or speech.

Although the differences in the duration estimates of speech and music have not been investigated before, a number of past studies have examined the effect of music expertise on the ability to discriminate differences in the duration of other stimuli, by either comparing the performance of musicians and non-musicians, or by taking into account the duration of the musical training. For example, Rammsayer and Altenmüller (2006) used a temporal discrimination task in which participants were presented with two intervals (that were either empty or contained white noise) and were asked to indicate which interval was longer. Results showed that a group of musically trained participants, which consisted of professional musicians and graduate music students, estimated the durations more accurately compared with a group of non-trained participants (Rammsayer & Altenmüller, 2006). Similarly, in a study we conducted to assess the effect of tempo of music excerpts on their perceived duration (Plastira & Avraamides, 2021), we observed a positive correlation between the time reproduction accuracy of the musically trained individuals and the duration of their musical training (Plastira & Avraamides, 2021).

In the current study, we revisit the issue of the duration of musical training and its relation to time estimation by using a time reproduction task with not only music but also speech stimuli. Testing a rather large sample of musically trained participants allowed us to use Structural Equation Modelling (SEM) and analyse data with Confirmatory Factor Analysis (CFA) to build a measurement Model of Auditory Time Perception. Our reasoning is that if time perception of auditory stimuli relies on a general process that deals with both speech and music stimuli, the same pattern of biases (i.e., overestimation/underestimation of stimuli’s duration) should be found for the two types of auditory stimuli and, consequently, a single-factor model will account for the data. Alternatively, if the perception of time relies on processes that are distinct for music and speech processing, different patterns should be found across the two types of stimuli and a two-factor model would provide a better fit for the data.

The purpose of our experiment was to explore the processes that underlie the perception of time and examine whether time perception of auditory stimuli relies on a general-purpose time perception mechanism or mechanisms that are distinct for speech and music. Furthermore, we investigated (1) the effect of the type and the duration of auditory stimuli on the reproduction of their duration and (2) the effect of musical training and rhythm perception on the perception of time.

## Methods

### Participants

One hundred and eleven undergraduate students from the University of Cyprus participated in the experiment in exchange for course credit. Only students who received formal musical training for at least 1 month any time during their life were eligible to register to the study. Fourteen participants reported that they did not receive any musical training, and they were excluded from the analyses. The final sample was, therefore, composed of 97 musically trained undergraduate students (72 female), aged between 18 and 38 years (*M* = 20.84, *SD* = 3.69).

### Materials

A time reproduction task was designed for the OpenSesame software (Mathôt et al., 2012) with music and speech audio files from our previous studies (Plastira &vraamides, 2020, 2021) used as stimuli. To create the speech tracks in our previous work, we recorded a professional musician reading a simple text while keeping the tone and the volume of her voice steady. The recorded speech was then segmented and the pieces were randomly blended. This procedure yielded an audio track with no comprehensible content that was then cut into pieces of various durations. To create the music tracks, a simple piano musical piece with a small pitch range, which was equivalent to that of the speech recording, was selected with the help of the professional musician. The musical piece was also segmented into different durations. For the purposes of the present study, we selected from the database of audio files speech and music clips with durations of 7, 8, and 9 s. All clips had identical volume. Seven different clips were used for each of the six unique combinations of type and duration. This resulted in a total of 42 different audio tracks presented in random order to each participant.

### Procedure

**Figure 1.**
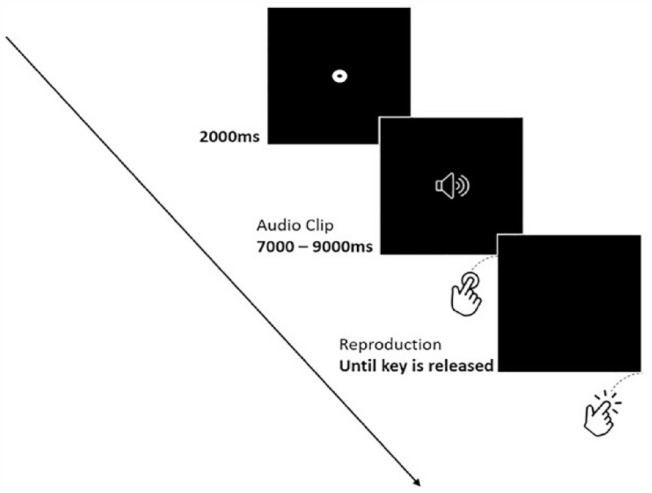
Schematic representation of a trial in Experiment 1.

Each participant was tested individually in a quiet laboratory. Participants were simply told that the study was about time perception with no other details provided. The experiment started with a practice phase containing three speech tracks and three music tracks, each lasting 5 s. Participants were instructed to listen to each track and after its completion to press the space bar for a duration equal to the duration of the track. A speaker icon was displayed on a black background during the presentation of each track (Figure 1). Once a track ended, the icon disappeared and the screen turned black, indicating to participants that they should begin reproducing the duration of the audio track by pressing the spacebar. After the end of the practice phase, the experimental phase began, which contained the 42 audio tracks that were selected for the experiment. As in the practice phase, the speaker icon appeared during the presentation of each track and disappeared after its completion. Participants were asked to press the spacebar, as soon as a track ended and to keep it pressed for a duration equal to that of the track. Two seconds after releasing the spacebar, the next audio file was presented and the same procedure continued until all stimuli were presented and their duration was reproduced. To isolate any extraneous auditory cues, participants listened to the stimuli through a pair of Sennheiser HD201 headphones. In addition, participants were instructed not to count during the presentation and the reproduction of the duration of the audio files, as past research indicates that this is the most efficient way to prevent a counting strategy during these tasks (Rattat & Droit-Volet, 2012).

After the completion of the experiment, participants were asked to fill out a short questionnaire (Plastira & Avraamides, 2021), which asked them to rate on 5-point Likert-type scale their competence to follow a musical rhythm. In addition, they were asked to provide information about the duration and type of their musical training.

#### Statistical analysis

To carry out the statistical analyses we first computed the average reproduced duration of every combination of audio type and duration for each participant. Next, we computed the reproduction ratio using the formula: reproduction ratio = reproduced duration/actual duration. Based on this formula, a ratio of 1 indicates a perfect reproduction of the actual duration, while ratios greater than 1 indicate overestimation and ratios lower than 1 indicate underestimation. Six reproduction ratio variables, corresponding to the reproductions of music stimuli of 7 s, 8 s, and 9 s, and speech stimuli of 7 s, 8 s, and 9 s, were used in the analysis. Each reproduction ratio variable was significantly correlated with all other reproduction ratio variables, with correlation coefficients ranging between .80 and .89 (Table 1). This strong and positive relationship between these variables suggests that participants who overreproduced the duration of one clip also tended to overreproduce the durations of the other clips. Similarly, for those underestimating a clip, there is a trend for consistent underestimation across all clips.

**Table 1.** Means, standard deviations, and Pearson correlations for the reproduction ratio of each duration and audio type combination.

Variables	*Mean*	*SD*	1	2	3	4	5	6
1. ratio_music7s	.75	.22	–					
2. ratio_music8s	.74	.21	.89**	–				
3. ratio_music9s	.73	.22	.89**	.88**	–			
4. ratio_speech7s	.86	.23	.82**	.86**	.80**	–		
5. ratio_speech8s	.82	.21	.82**	.87**	.84**	.85**	–	
6. ratio_speech9s	.78	.21	.82**	.86**	.86**	.88**	.88**	–

*SD*: standard deviation.

* *p ⩽ 0.01.

Analyses were carried out using the IBM SPSS Statistics 25 IBM Corp, 2017) and the JASP statistical software version 0.16.1 (JASP Team, 2022). For the main analysis, two competing measurement models were constructed, to examine, through CFA, whether the six observed reproduction ratio variables of Table 1 were related to a single latent variable (Auditory Time Perception) or to two latent variables (Music Time Perception and Speech Time Perception). Then, we used SEM to assess the effect of months of musical training on time perception. The CFA and the SEM analyses were conducted using the maximum likelihood estimation method. Before building the models, the Mardia’s test was used to assess the assumption of multivariate normality. The test showed that the assumption was violated, χ^2^(120) = 244.33, *p* < .001, hence, the analyses were performed using robust maximum likelihood estimation with Mplus emulation in JASP (JASP Team, 2022).

Furthermore, a repeated measures Analysis of Variance (ANOVA) was carried out to examine the effects of audio type and duration on reproduced durations. Finally, correlational analyses were performed to examine the relation between the reproduction ratio, the duration of musical training, and the self-reported ability to follow musical rhythm.

#### Measurement and structural models

The fit of each model was assessed with the following goodness-of-fit indices and the following cutoff criteria: chi-square for which a *p* > .05 indicates a good fit, Goodness-of-fit Index (GFI), Comparative Fit Index (CFI) with values ⩾. 90 demonstrating acceptable fit and ⩾.95 demonstrating a good fit; Tucker–Lewis index (TLI) which shows acceptable fit for values less than 1 but greater than .90, and good fit for values ⩾.95; Standardised Root Mean Squared Residual (SRMR) showing good fit for values ⩽.08; Root Mean Square Error of Approximation (RMSEA) showing acceptable fit if ⩽.08 and good fit when ⩽.06, and closeness of fit (PCLOSE) with value >.50 showing high probability for RMSEA to be <.05 (Byrne, 2016; Kline, 2005; Schneider et al., 2015). Finally, the Akaike Information Criterion (AIC) and the Bayesian Information Criterion (BIC) indices were used to assess model fit. Low AIC and BIC values indicate better fit of a model to the data compared with a model with higher values (Burnham & Anderson, 2004).

## Results

### Measurement model. CFA

**Figure 2.**
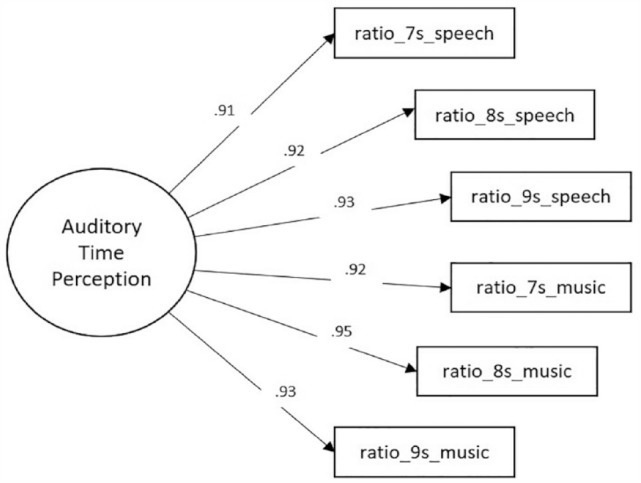
Single-factor measurement model with standardised estimates. This CFA model examined the relationship of the six observed variables and a latent variable named Auditory Time Perception.

A CFA was conducted, using the robust maximum likelihood estimation method with Mplus emulation, to test a measurement model examining the relationship of the six observed variables and a latent variable named Auditory Time Perception (Single-factor Model) (Figure 2).

**Figure 3.**
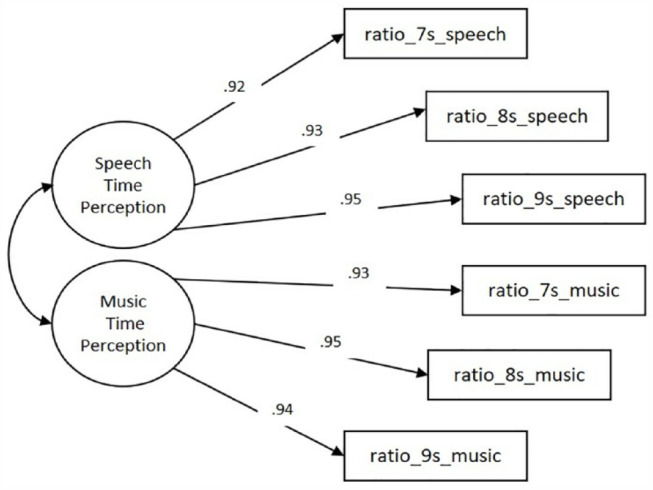
Two-factor measurement model with standardised estimates. This CFA model examined the relationship of the six observed variables and two latent variables named Music Time Perception and Speech Time Perception.

As shown in Table 2, not all fit indices were in the acceptable range for the Single-factor Model. Hence, a two-factor measurement model (Two-factor Model) was examined, with each factor having three indicators (Figure 3). The reproduction ratio of the speech tracks were the indicators for the latent variable Speech Time Perception, whereas the reproduction ratio of the music tracks were the indicators for the latent variable Music Time Perception. The two latent factors were correlated. Fit indices showed that the Two-factor Model had a better fit to the data than the Single-factor Model (Table 2).

**Table 2.** Fit indices values for the measurement models estimated with the robust maximum likelihood method with Mplus emulation on JASP.

Model	$x^{2}$	df	GFI	CFI	SRMR	TLI	RMSEA	PCLOSE	AIC	BIC
Single-factor Model	38.429* (*p* < .001)	9	.980	.963	.018	.939	.184	RMSEA*p*-value 2.131e-4	-884.485	-838.140
Two-factor Model	18.498 (*p* = .02)	8	.991	.987	.012	.976	.116	RMSEA*p*-value .058	-902.416	-853.496

GFI: Goodness-of-fit Index; CFI: Comparative Fit Index; SRMR: Standardised Root Mean Squared Residual; TLI: Tucker–Lewis index; RMSEA: Root Mean Square Error of Approximation; PCLOSE: closeness of fit; AIC: Akaike Information Criterion; BIC: Bayesian Information Criterion.

### Robust maximum likelihood estimation on JASP

In addition, all item factor loadings of the Two-factor Model were significant (*p* < .001), ranging from .92 to .95 for the factor Speech Time Perception and from .93 to .95 for the factor Music Time Perception. The correlation between the factors Speech Time Perception and Music Time Perception was very high, .96 (*p* < .001).

### Structural model. The effect of musical training

**Figure 4.**
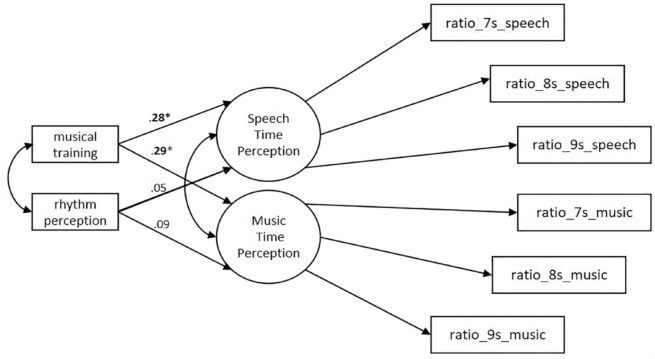
Structural model: this model examined the effect of duration of musical training (in months) and of rhythm perception (self-reported) on Speech Time Perception and Music Time Perception. In this model the duration of musical training and rhythm perception correlate.

To examine the effects of the duration of musical training on Speech Time Perception and on Music Time Perception, a structural model was tested. The duration of the musical training of the participants, that ranged from 1 to 228 months (*M* = 45.99, *SD* = 40.87) and the self-reported rhythm perception (*M* = 2.95, *SD* = 1.02), were added in the Two-factor Model, as predictors for both latent variables (Figure 4). The duration of musical training was significantly correlated with the self-reported rhythm perception, Spearman’s rho = .37, bias-corrected and accelerated bootstrap 95% confidence interval (BCa 95% CI = [.18, .54]), *p* < .001. The results of the structural model indicated that the duration of musical training had a significant positive effect on the factor Speech Time Perception (β = .28, *p* = .01) and on the factor Music Time Perception (β = .29, *p* = .02). On the contrary, self-reported rhythm perception did not have a significant effect either on Speech Time Perception (β = .05, *p* = .65) or on Music Time Perception (β = .09, *p* = .37).

Removing rhythm perception from the structural model yielded results that were similar to those of the structural model that included both predictors. There was still a significant effect of the musical training on the factor Speech Time Perception (β = .31, *p* = .003) as well as on the factor Music Time Perception (β = .31, *p* = .006).

### Repeated measures ANOVA

To examine the effect of the duration and the type of audio tracks on subjective time perception, we conducted a two-way repeated measures ANOVA. Results revealed significant main effects for duration, *F*(2,192) = 21.62, *p* < .001, *η_p_*^2^ = .18 and audio type, *F*(1,96) = 81.32, *p* < .001, *η_p_*^2^ = .46. The interaction between duration and audio type was also significant. Due to the violation of the normality assumption for this interaction, as revealed by the Mauchly’s test for sphericity, the Greenhouse–Geisser correction was applied, *F*(1.883, 180.803) = 7.02, *p* = .001, *η_p_*^2^ = .07.

Concerning the main effect of duration, results showed that the longer the actual duration of the audio track was, the more it was underestimated. Within-subjects contrasts revealed a significant difference between the reproduction ratio of audio tracks with a duration of 7 s and the reproduction ratios of the two other durations (7 s vs. 8 s: *p* < .001 and 7 s vs. 9 s: *p* = .001). In addition, the difference between the reproduction ratio of 8 s and 9 s audio tracks was also significant, *p* = .001.

Regarding the effect of the type of audio track, results revealed that the average reproduction ratio of the speech tracks was significantly greater than the average reproduction ratio of the music tracks. In other words, the speech tracks were overall reproduced as longer and more accurately, compared with the music tracks.

**Figure 5.**
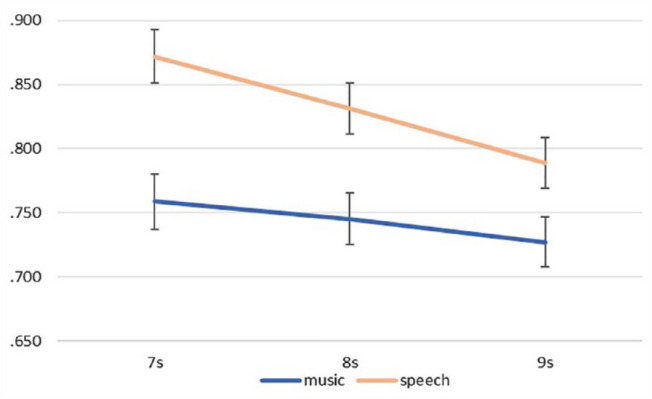
Mean reproduction ratio as a function of actual duration (7–9 s) and audio type.

As seen in Figure 5, the interaction was driven by the presence of greater differences across the three different durations of the speech tracks compared with those across the three different durations of the music tracks. More specifically, the difference between the reproduction ratios of the speech tracks of 7 s and 9 s was significantly greater compared with the difference of the ratios of the music tracks of 7 s and 9 s, *F*(1,96) = 17.14, *p* < .001, η_p_^2^ = .15.

### Repeated measures Analysis of Covariate (ANCOVA)

A repeated measures ANCOVA was also performed, controlling for the effect of the duration of musical training and self-reported rhythm perception. The covariates of musical training and musical rhythm were mean centred across all participants (Schneider et al., 2015). The Mauchly’s test for sphericity indicated that the assumption of normality for the duration × audio type interaction was violated, hence the Huynh-Feldt correction was applied. The results of the repeated measures ANCOVA indicated that there were significant main effects for duration, *F*(2,188) = 21.24, *p* < .001, η_p_^2^ = .18 and audio type, *F*(1,94) = 80.39, *p* < .001, η_p_^2^ = .46 and a significant duration × audio type interaction, *F*(1.96,184.05) = 6.93, *p* = .001, η_p_^2^ = .07 (Huynh-Feldt corrected). Results also showed that there was a significant effect of the duration of musical training covariate, *F*(1,94) = 7.15, *p* = .009, η_p_^2^ = .07, but no significant effect of the musical rhythm covariate, *F*(1,94) = .483, *p* = .49, η_p_^2^ = .01.

Overall, the results from the ANCOVA showing that there were significant main effects and an interaction of audio type and duration even after controlling for musical training and musical rhythm suggest that the findings were specific to the characteristics of the stimuli and not due to the skills of the participants.

### Correlational analyses

Correlational analyses were performed to examine the relation between the mean reproduction ratio, the duration of musical training (in months), and the self-reported sense of musical rhythm. As already stated, the duration of musical training was significantly correlated with the self-reported sense of musical rhythm. Results also showed that the mean reproduction ratio was significantly correlated with the duration of musical training, *r* = .31, BCa 95% CI = [.13, .49], *p* = .002. The duration of musical training also correlated significantly with both the mean reproduction ratio for speech, *r* = .30, BCa 95% CI = [.11, .46], *p* = .003, and the mean reproduction ratio for music stimuli, *r* = .31, BCa 95% CI = [.12, .49], *p* = .002. However, the correlation between the mean reproduction ratio and the self-reported sense of musical rhythm was not significant, rho = .18, BCa 95% CI = [˗.02, .36], *p* = .08. Finally, the self-reported sense of musical rhythm did not correlate significantly with the mean reproduction ratio for either speech, rho = .10, BCa 95% CI = [.01, .38], *p* = .06 or music stimuli, rho = .17, BCa 95% CI = [˗.03, .37], *p* = .09.

## Discussion

The present study is the first that attempted to build a time perception model for musically trained individuals. To this end, we used a time reproduction task with both music and speech-like stimuli. Of great interest was also the examination of the effects of the duration of musical training and of rhythm perception on time estimation as well as the effects of music and speech on the perception of time.

First, to examine whether there is a general Auditory Time Perception or two audio-type-specific dimensions of time perception, we estimated measurement and structural models. To our knowledge, no previous study has attempted to explore these dimensions of time perception, although a number of studies have assessed the relation between speech and music processing and their underlying mechanisms (Albouy et al., 2020; Koelsch & Siebel, 2005; Patel, 2003; Yu et al., 2017).

Our results showed that a Two-factor Model had a better fit to the data than a Single-factor Model. For the Two-factor model, the three indicators of each of the two latent factors (Speech Time Perception and Music Time Perception) reflected its underlying construct and the factor loadings were all significant. We note, however, a very strong latent association between the two factors. As no previous research has examined a time perception model using speech and music stimuli, the rationale of moving from the Single-factor Model to the Two-factor Model was based on the model fit, on the fact that the items could be separated into two groups because they were indeed reflecting the perception of the duration of two different audio types, and on findings that different structures underlie the cognitive processing of music and speech (Asaridou & McQueen, 2013; Besson et al., 2011). The two dimensions of Auditory Time Perception support the idea that time is experienced differently and may be processed by separate, but highly correlated, mechanisms for speech and music. Past research has shown that neural structures responsible for speech and music processing are at least partially overlapping (Koelsch & Siebel, 2005; Patel, 2003; Rimmele et al., 2021). Furthermore, although music and speech are two qualitatively different auditory stimuli, they are both crucial means of communication and share acoustic features, like the pitch, the tempo, and the rhythm, that affect the way the communicated information is perceived. All things considered, it is possible that the relation between Music Time Perception and Speech Time Perception is mediated by the commonalities in both the features and the processing of the two audio types, even though participants in the current study were instructed to pay attention to the duration of the stimuli and not their type.

Our findings also revealed a significant main effect and an interaction between actual duration and audio type, even when controlling for the effect of the duration of musical training and the self-reported rhythm perception. More specifically, the results revealed that as the actual duration of the audio tracks increased, the accuracy of the reproduced durations diminished. This result appears to support the notion that there is a greater underestimation for the duration of long compared with short stimuli (Brown, 1995; Lejeune & Wearden, 2009), a pattern that displays a quasi-Vierordt’s effect. Based on Vierordt’s law, short durations are overestimated, and long durations are underestimated. Our results demonstrate that although all our stimuli were underestimated, this underestimation became more pronounced as the actual duration lengthened, exhibiting a Vierordt-like pattern. This duration effect was also observed in our previous studies that used speech stimuli (Plastira & Avraamides, 2020) or music stimuli (Plastira & Avraamides, 2021).

Furthermore, our results showed that there are differences in the way speech and music tracks are reproduced. Although the durations of speech and music tracks were overall undereproduced, the duration of the speech tracks was reproduced as longer than that of the music tracks. That is, speech tracks were reproduced more accurately compared with the music tracks. Previous studies that examined the effect of the presence of music in real-life situations, like in a waiting room environment or during an on-hold telephone queue, showed that a waiting time that includes music is judged as shorter compared with an empty waiting time (Guéguen & Jacob, 2002; North & Hargreaves, 1999). Our results extend these previous findings by demonstrating that music tracks are reproduced as shorter compared with other types of stimuli as well.

One potential explanation for this finding is that less attention is focused on the timing process when music is played. In a past study, North and Hargreaves (1999) had participants estimate the duration of the time they waited in a laboratory for an experiment to start, when music or nothing was playing in the background. The results showed that the waiting time was underestimated more when music was playing, compared with the no-music waiting time (North & Hargreaves, 1999). To interpret their results, the authors referred to the Attentional Gate Model and the internal mechanism that modulates human time perception, i.e., the internal clock (Droit-Volet & Meck, 2007). Based on these models, time is processed through three stages: a pacemaker that produces pulses, a gate that controls the flow of pulses, and an accumulator that registers the number of pulses that pass through. The accumulated number of pulses corresponds to the perceived duration, such that more pulses lead to longer perceived durations. According to the Attentional Gate Model, attention plays a crucial role in the accumulation of pulses and thus in the perception of time duration. More specifically, the opening of the gate between the pacemaker and the accumulator of the internal clock can be modulated by attentional resources. When attention is directed towards the passage of time, the gate opens wider, allowing more pulses to accumulate and resulting in a longer perceived duration. On the contrary, when attention is directed towards external stimuli, the gate narrows, resulting in the accumulation of fewer pulses and a shorter perceived duration. Based on this, North and Hargreaves (1999) argued that the attentional resources were focused on the time during the no-music waiting time and due to that, its duration was experienced as longer, compared with that of the music waiting time, during which the attention was focused on the music and away from time (North & Hargreaves, 1999). These results suggest that maybe in our study too, participants underestimated more the duration of the music stimuli because they were more attentional capturing than the speech stimuli. The fact that music tracks are judged as shorter in duration than other types of sounds was also shown in the study of Droit-Volet et al. (2010), who suggested that there is a relation between the melodic nature of music and the experience of the passage of time. In that study, music tracks played in minor and major key were both perceived as shorter in duration compared with control sine wave stimuli.

In spite of what has been reported before, in the comparison of reproduction accuracy of music versus speech stimuli, one would expect the musically trained participants in the current study to be more accurate at reproducing the duration of the former than the latter due to their experience with music reproduction. However, this was not the case. A possible explanation is that participants were just asked to reproduce the duration of the music tracks by pressing a key, following a procedure that does not correspond to the routines related to the musical training. Perhaps if they were asked to reproduce the tracks by playing them on a musical instrument they would perform more accurately. In addition, it’s possible that musically trained individuals are more accustomed to estimating and reproducing the duration of individual notes rather than the overall duration of a musical piece. This could explain why their performance on reproducing the duration of music tracks here did not surpass their performance on reproducing the duration of speech tracks. Overall, it seems that the expertise cannot explain the differences we observed in the reproduced durations of music versus speech.

In addition, it is worth considering the potential influence of cognitive load caused by the exposure to the scrambled speech and to the music stimuli. It is possible that, in the present study, participants actively searched for meaning in the scrambled speech clips, experiencing a higher cognitive load compared with the music stimuli. As previous research has shown, increased cognitive load can lead to shorter perceived durations (Ahn et al., 2006; Block & Gellersen, 2010; Khan et al., 2006). However, our results showed that the music stimuli were estimated as shorter than the speech stimuli, suggesting that if there was any effect of cognitive load, it was masked by the effects of other factors with stronger influence on time estimation.

A potential account for the different duration estimates for speech and music is that there were differences in emotional characteristics across the two types of stimuli. More specifically, one possibility is that the greater underestimation of music than speech stimuli is linked to differences in elicited arousal by the two stimuli. Indeed, previous studies that assessed the effect of various stimulus properties in time estimation tasks, such as the music tempo (Droit-Volett al., 2013; Plastira & Avraamides, 2021) and the speed of speech (Plastira &vraamides, 2020), suggest that subjective arousal induced by these features may influence the way the duration of the stimuli is perceived. Subjective arousal is typically believed to have an effect on the pacemaker of the internal clock (Droit-Volet et al., 2013). More specifically, it is suggested that high subjective arousal speeds up the pacemaker of the internal clock leading to the accumulation of more pulses. Based on the internal clock model, the number of accumulated pulses defines the perceived duration of a stimulus (Zakay & Block, 1995). That is, if the presence of an arousing stimulus increases the number of accumulated pulses, then its duration will be estimated as longer than that of a non-arousing stimulus (Droit-Volet et al., 2013; Droit-Volet & Meck, 2007; Schwarz et al., 2013).

The feelings of pleasantness induced by a stimulus presentation are also shown to have an effect on the perception of time. In fact, the levels of pleasantness induced during an experienced duration are linked to the amount of attention allocated to time (Droit-Volet et al., 2013). An unpleasant or not very pleasant stimulus directs attention to the passage of time, leading to a wide opening of the attentional gate, the accumulation of more pulses, and a longer perceived duration. On the contrary, a pleasant, attentional capturing stimulus appears to narrow the attentional gate, resulting in a reduced accumulation of pulses and, consequently, shorter perceived durations.

To examine whether our stimuli would induce different arousal and pleasantness feelings depending on their characteristics, two follow-up experiments were conducted and are presented in the online Supplementary Material. In these experiments, an arousal rating task and a pleasantness rating task were administered to two groups of participants (*n* = 16 and *n* = 13, respectively). The results showed that music tracks were rated as less arousing and more pleasant compared with the speech tracks. Thus, results from these additional experiments seem to support the aforementioned theories and a possible relation between time estimates, arousal, and pleasantness ratings given for qualitatively different stimuli.

Our findings from the main experiment described here also indicated that there were greater differences between the reproduction accuracy of the shortest (7 s) and longest (9 s) durations for the speech compared with the music audio tracks, although speech tracks were reproduced significantly more accurately than music tracks. Maybe the familiarity of musically trained participants with reproducing music durations made their reproductions of music tracks less prone to the increase of the actual duration, compared with speech tracks. This possibility can be explored further by experiments with longer actual durations of both music and speech tracks.

The present study also examined the relation between the duration of musical training, rhythm perception, and the perception of time. Previous studies also assessed the relation between musical training and time perception, with the use of different stimuli and tasks. Marie et al. (2012), e.g., showed that musically trained individuals are better than non-trained individuals at detecting duration differences, when comparing harmonic sounds of different durations with standard ones. Similarly, Rammsayer and Altenmüller (2006) used a temporal discrimination task and showed that musicians are more accurate at estimating the durations of empty or white-noise intervals, compared with non-musicians (Rammsayer & Altenmüller, 2006). Furthermore, in our previous study where we assessed the perception of time of musically trained and non-trained participants with a time reproduction task that contained music stimuli of various speeds and durations (Plastira &vraamides, 2021), we found a positive relation between reproduction accuracy and the duration of musical training, as well as between reproduction accuracy and the self-reported ability to follow a musical rhythm.

In the present study, we aimed to extend past results by testing a large sample of musically trained participants. When we examined the effects of musical training and rhythm perception on the two latent factors, Music Time Perception and Speech Time Perception, we found that the duration of musical training had an effect on both Music and Speech Time Perception, whereas self-reported rhythm perception had no effect. Our results also showed that although the duration of musical training had a significant effect on the reproduction ratio, rhythm perception did not. The absence of an effect of rhythm perception may be attributed to that the sample included only musically trained individuals who generally reported high rhythm perception, restricting thus variability in this measure.

Notably, the duration of musical training related to rhythm perception. This relation is described as bidirectional in the literature (Hambrick et al., 2016; Moreno et al., 2009; Schellenberg, 2015). On one hand, as musical training increases, it enhances rhythm perception skills. On the other hand, good rhythm perception skills can cause people to take up or continue taking music lessons.

Correlational analyses also indicated that individuals with longer musical training were also more accurate in their reproductions, compared with the individuals with shorter musical training. Nevertheless, in contrast to our previous work with only music stimuli (Plastira &vraamides, 2021), in the present study in which participants reproduced both music and speech, there was no significant correlation between self-reported rhythm perception and reproduction accuracy. Overall, these results suggest that maybe the relation between rhythm perception and time perception is stronger when participants are exposed to music alone, than when exposed to speech alone or to both speech and music. To further examine this, in the present study we also investigated the relation between rhythm perception and the reproduction accuracy for music and speech, separately. The results showed that neither the reproduction accuracy of music, nor the reproduction accuracy of speech related to rhythm perception. Therefore, the differences in the results may be attributed to the fact that stimuli of different durations were used in the present study, compared with those used in the previous studies. Here, we only used audio files of three durations, 7 s, 8 s, and 9s, whereas previous studies used audio files of 2 s–8 s. Hence it may be the case that the relation between rhythm perception and reproduction accuracy is stronger for short durations, especially as far as music is concerned. Our results suggested that people with superior rhythm perception are more accurate than those with inferior rhythm perception at reproducing short but not long musical pieces. One explanation for this is that following a musical rhythm accurately requires remembering short musical units. Finally, the absence of a relation between the reproduction accuracy of speech and the rhythm perception could be explained by that speech tracks do not consist of rhythmic components, tones, and beats that relate to the perception of rhythm.

In summary, in the present study we built for the first time a time perception model for musically trained individuals and provided evidence that different mechanisms can be responsible for perceiving and estimating the duration of music and speech. Moreover, we explored how musically trained individuals perceive time and we showed that their time estimation accuracy related to the duration of their musical training. Our results from the first experiment showed that the duration speech is more accurately reproduced compared with music. Our supplemental experiments explored possible causes for these differences and suggested that higher subjective arousal and lower pleasantness for speech stimuli could explain this pattern of results. Because in the present study we used scrambled speech, a future study may compare the effects of music and actual speech on the perception of time and examine whether the effects of the two types of stimuli are similar. Moreover, to enhance the validity and generalisability of our results, future studies may use speech and music stimuli that are matched for arousal and pleasantness, based on ratings by a larger sample of participants. This would allow for a more comprehensive examination of the temporal processing of speech and music, of its relation with their emotional characteristics, and of how the durations are reproduced by both trained and non-trained individuals.

In conclusion, this study’s most notable discovery is that the processing of the duration of music and speech stimuli seems to rely on separate but related mechanisms. These mechanisms may be attributed to the unique acoustic and rhythmic characteristics of each stimulus, which influence their temporal processing. The presence of distinct regions in the brain for speech and music processing (Albouy et al., 2020;idtis & Volpe, 1988) support the idea of separate mechanisms. On the contrary, the relationship between the two mechanisms could be attributed to the fact that there is a partial overlap in the brain regions engaged in the processing of speech and music (Koelsch & Siebel, 2005; Patel, 2003; Rimmele et al., 2021). Consequently, the findings of this study also support the idea that the processing of speech and music are interrelated and also connected to a general auditory processing ability (Asaridou & McQueen, 2013). Our results also extend past research on the role of domain expertise in time perception and they can be used as a basis for future studies on the topic.

## Supplemental Material

sj-docx-1-qjp-10.1177_17470218231205857 – Supplemental material for Music and speech time perception of musically trained individuals: The effects of audio type, duration of musical training, and rhythm perception Supplemental material, sj-docx-1-qjp-10.1177_17470218231205857 for Music and speech time perception of musically trained individuals: The effects of audio type, duration of musical training, and rhythm perception by Miria N Plastira, Michalis P Michaelides and Marios N Avraamides in Quarterly Journal of Experimental Psychology
