# Supplementary material for: Music and speech time perception of musically trained individuals: The effects of audio type, duration of musical training, and rhythm perception
Source: Q J Exp Psychol (Hove). 2023 Oct 28;77(9):1835–45. doi: 10.1177/17470218231205857 (PMC11373153; doi:10.1177/17470218231205857)
Supplement: sj-docx-1-qjp-10.1177_17470218231205857 – Supplemental material for Music and speech time perception of musically trained individuals: The effects of audio type, duration of musical training, and rhythm perception [file sj-docx-1-qjp-10.1177_17470218231205857.docx]

Supplementary Material for:

**Music and Speech Time Perception of Musically Trained Individuals: The Effects of Audio Type, Duration of Musical Training and Rhythm Perception.**

Miria N. Plastira^1,2^ , Michalis P. Michaelides^1^ and Marios N. Avraamides^1,2^

^1^Department of Psychology, University of Cyprus

^2^ CYENS Centre of Excellence, Nicosia, Cyprus

**Author Note**

Miria N. Plastira <https://orcid.org/0000-0002-4017-078X>

Michalis P. Michaelides https://orcid.org/0000-0001-6314-3680

Marios N. Avraamides <https://orcid.org/0000-0002-0049-8553>

Correspondence concerning this article should be addressed to Miria N. Plastira. E-mail:

[miria-90@hotmail.com](mailto:miria-90@hotmail.com)

To examine whether subjective arousal may account for the results of our main Experiment, we carried out a follow-up experiment in which we asked new participants to rate their arousal for each stimulus used in the main Experiment.

**Experiment 2**

The purpose of Experiment 2 was to examine the hypothesis that differences in subjective arousal can account for the effect of audio type on the reproduction of time that was found in our main Experiment. For this experiment, new participants were asked to rate the music and speech audio tracks used in the main Experiment of the study, in terms of the subjective arousal they caused them.

**Participants**

Sixteen participants aged from 18 to 50 (M= 27.88, SD=8.60) were asked to rate the audio tracks.

**Materials**

A new task was designed using the OpenSesame software (Mathôt, Schreij, & Theeuwes, 2012) where the 21 music audio files and the 21 speech audio files that were used in the main experiment were presented to the participants in random order. The task entailed rating each audio file on a 0-9 scale, in terms of subjective arousal, using the number pad of a keyboard. No estimates of duration were made.

**Procedure**

The experiment started with a practice phase with two 5s speech tracks and two 5s music tracks. The experimental phase followed, which contained the 42 audio tracks that were used in the previous experiment. In both phases, after the end of each presented audio track participants were asked to rate it in terms of the psychological arousal it caused them. Each audio track was presented two seconds after the previous rating was provided by the participant. As in our main Experiment, participants listened to the audio tracks through the same headphones.

**Results**

Results showed a main effect for audio type with speech tracks being evaluated as more arousing than music tracks, F(1,15) = 214.72, p = .005, *η_p_^2^* = .42. Neither a main effect for duration nor an audio type x duration interaction were observed.

*Figure 6 Mean* subjective arousal ratings as a function of actual duration (7s-9s) and audio type.

**Discussion**

The results of Experiment 2 showed that no significant differences were observed between the subjective arousal caused by short and long tracks. However, speech tracks were rated as more arousing compared to the music tracks. This is not surprising given that speech and music have distinct features. The basic sound units of speech and music, that is phonemes and pitches respectively, are qualitatively different (Brown, S., 2000). The fact that multiple sets of phonemes are combined to create speech and multiple sets of pitches and note lengths are combined to create music (Brown, 2000; Wolfe, 2002) makes it clear that there are fundamental acoustic differences that can affect differently the subjective arousal of a listener.

Our findings are in line with the explanation that the reason speech stimuli were reproduced as longer in duration, in our main Experiment with the reproduction task, compared to music stimuli, is that they were more arousing. Past studies show that arousal affects the function of the internal clock and, consequently, distorts the perception of time (Droit-Volet et al., 2013; Droit-Volet & Meck, 2007; Schwarz et al., 2013). More pulses are emitted and accumulated during the presentation of an arousing stimulus than during the presentation of a less arousing stimulus.

Beyond arousal, the pleasantness of a stimulus is also believed to have an effect on the perception of time, by modulating the function of a different component of the internal clock (Droit-Volet et al., 2013). This component is the gate of the clock which, based on the Attentional Gate model, is located between the peacemaker and the accumulator and it determines the number of pulses that pass to the accumulator (Zakay & Block, 1995). The opening of the gate is controlled by the attentional resources that are allocated on the passage of time. The gate opens more when the attention is focused on the passage of time, thus more pulses are accumulated, compared to when the attention is focused away from time and on an external stimulus. Hence, a duration is estimated as longer in the first case, compared to the latter. Pleasantness levels are shown to be related to the amount of attention that is allocated to the time (Droit-Volet et al., 2013). A pleasant stimulus seems to capture the attention of the participants making the opening of the gate to narrow and causing the accumulation of fewer pulses and the underestimation of the duration. On the other hand, an unpleasant stimulus shifts the focus of the attention on the passage of time, causing a further opening of the gate leading to the accumulation of more pulses. Hence, the duration of an unpleasant stimulus is perceived as longer than that of a pleasant stimulus (Droit-Volet et al., 2013). Considering that pleasantness seems to be a factor that affects the perception of time, Experiment 3 was conducted to assess whether music and speech tracks induce different levels of pleasantness, which could explain the differences in time estimation.

**Experiment 3**

The purpose of Experiment 3 was to assess the pleasantness levels of our audio stimuli, a factor that has been shown in the past (Droit-Volet et al., 2013) to influence time perception. To that end a new group of participants rated the music and speech audio tracks used in our main Experiment, on a pleasantness scale.

**Participants**

Thirteen participants aged from 21 to 40 (M= 28.25, SD=6.80) rated the audio tracks.

**Materials**

The rating task used in Experiment 3 was the same as that of Experiment 2, with the exception that the arousal scale was modified to a pleasantness scale. As in Experiment 2, the stimuli used in the task were presented to the participants in random order and they comprised of the 21 music audio files and the 21 speech audio files that were used in our reproduction Experiment.

**Procedure**

The procedure of the experiment was identical to that of Experiment 2, except that participants were asked to rate the stimuli on a 0-9 scale, in terms of their pleasantness. The scale ranged from unpleasant to pleasant. It was clarified to participants that they shouldn’t assess whether they liked the audio tracks or not. Instead, they were instructed to rate them solely based on how pleasant they found their sound.

**Results**

Results showed a main effect for audio type F(1,12) = 97.41, p < .001, *η_p_^2^* = .89 with music tracks being evaluated as more pleasant than speech tracks and a main effect for duration, F(2,24) = 3.86, p = .04, *η_p_^2^* = .24, with pleasantness ratings getting higher as the duration of the tracks increased (Figure 7). Within-subjects contrasts showed that the only significant difference in the overall pleasantness ratings was between the shortest (7s) and the longest (9s) tracks, with the longest tracks being rated as more pleasant compared to the shortest ones (M=4.08 and M=4.32, respectively). Importantly, results revealed a significant duration x type interaction, F(2,24) = 4.24, p = .03, *η_p_^2^* = .26. Within-subjects contrasts revealed that the difference between the longest (9s) and the shortest tracks (7s) was greater for the music audio tracks than the speech audio tracks (p = .03). However, it should be noted that the long music tracks were rated as more pleasant (M=7.34), than the short music tracks (M=6.86), while the long speech tracks were rated as slightly less pleasant (M=1.30) compared to the short speech tracks (M=1.31).

Figure 7 Mean subjective valence ratings as a function of actual duration (7s-9s) and audio type**.**

**Discussion**

The results of Experiment 3 showed that the pleasantness ratings for music and speech tracks differed significantly. The fact that music was judged as more pleasant, compared to speech, was expected, since music is usually linked with pleasant experiences (Idrobo-Avila et al., 2018). However, in our case the results may also be attributed to the fact that the speech tracks used here consisted of pseudowords as opposed to actual speech, while music consisted of piano melody, a sound that is more familiar to the human ear. The fact that longer music tracks were judged as more pleasant compared to shorter ones, may also be associated with the calming attributes of piano melody. The longer the presentation of the melody, the more pleasant were the feelings of the participants. On the other hand, the pleasantness ratings for the speech tracks decreased slightly as its duration increased. This may also be attributed to the use of speech-like stimuli as opposed to real speech. Nonetheless, the difference between the ratings of long and short tracks was greater for the music tracks, compared to that of the speech tracks, suggesting that the effect of duration on pleasantness was more salient for the music than for the speech tracks.

In addition to arousal, the pleasantness ratings of our stimuli can also explain the results of our main Experiment regarding the effect of audio type. That is, that listening to music was judged as more pleasant compared to listening to the speech stimuli in this experiment, may provide a plausible explanation as to why music stimuli were reproduced as shorter than speech stimuli in the main Experiment. More specifically, by being more pleasant, the music stimuli might have captured the attention of the listeners and narrowed the opening of the attentional gate. As a result, music was experienced as shorter than speech.

References

Brown, S. (2000). The "musilanguage" model of music evolution. In N. Wallin, B. Merker & S. Brown (Eds.), *The origins of music* (pp. 271). MIT Press.

Droit-Volet, S., Ramos, D., Bueno, J. L., & Bigand, E. (2013). Music, emotion, and time perception: the influence of subjective emotional valence and arousal? *Frontiers in Psychology, 4*, 19-30. <https://doi.org/10.3389/fpsyg.2013.00417>

Droit-Volet, S., & Meck, W. H. (2007). How emotions colour our perception of time. *Trends in Cognitive Sciences, 11*(12), 504-513. <https://doi.org/10.1016/j.tics.2007.09.008>

Idrobo-Avila, E. H., Loaiza-Correa, H., Van Noorden, L., Munoz-Bolanos, F. G., & Vargas-Canas, R. (2018). Different Types of Sounds and Their Relationship With the Electrocardiographic Signals and the Cardiovascular System–Review. *Frontiers in Physiology, 9*, 525. <https://doi.org/10.3389/fphys.2018.00525>

Mathôt, S., Schreij, D., & Theeuwes, J. (2012). OpenSesame: An open-source, graphical experiment builder for the social sciences. Behavior research methods, 44, 314-324.

Schwarz, M. A., Winkler, I., & Sedlmeier, P. (2013). The heart beat does not make us tick: The impacts of heart rate and arousal on time perception. *Attention, Perception, & Psychophysics, 75*(1), 182-193. <https://doi.org/10.3758/s13414-012-0387-8>

Wolfe, J. (2002). Speech and music, acoustics and coding, and what music might be ‘for’. Paper presented at the *Proc. 7th International Conference on Music Perception and Cognition,* 10-13.

Zakay, D., & Block, R. A. (1995). An attentional-gate model of prospective time estimation. In M. Richelle, V. D. Keyser, G. d'Ydewalle & A. Vandierendonck (Eds.), *Time and the dynamic control of behavior* (pp. 167-178). Universite de Liege Liège, Belgium.
